# Optically Controllable 2D Material/Complex Oxide Heterointerface

**DOI:** 10.1002/advs.202002393

**Published:** 2020-08-20

**Authors:** Tao Liu, Cheng Han, Du Xiang, Kun Han, Ariando Ariando, Wei Chen

**Affiliations:** ^1^ SZU‐NUS Collaborative Innovation Center for Optoelectronic Science & Technology International Collaborative Laboratory of 2D Materials for Optoelectronics Science and Technology of Ministry of Education Institute of Microscale Optoelectronics Shenzhen University Shenzhen 518060 P. R. China; ^2^ Department of Chemistry National University of Singapore 3 Science Drive 3 Singapore 117543 Singapore; ^3^ Department of Physics National University of Singapore 2 Science Drive 3 Singapore 117542 Singapore; ^4^ Joint School of National University of Singapore and Tianjin University International Campus of Tianjin University Binhai New City Fuzhou 350207 P. R. China; ^5^ National University of Singapore (Suzhou) Research Institute 377 Lin Quan Street, Suzhou Industrial Park Suzhou Jiangsu 215123 P. R. China

**Keywords:** amorphous strontium titanium oxide (a‐STO), MoTe_2_/a‐STO heterostructures, optically controllable interfacial states, polarity tunable photoresponses

## Abstract

Heterostructures play a vital role in functional devices on the basis of the individual constituents. Non‐conventional heterostructures formed by stacking 2D materials onto structurally distinct materials are of great interest in achieving novel phenomena that are both scientifically and technologically relevant. Here, a heterostructure based on a 2D (molybdenum ditelluride) MoTe_2_ and an amorphous strontium titanium oxide (a‐STO) thin film is reported. The heterostructure functions as a high‐performance photodetector, which exhibits anomalous negative photoresponse in the pristine device due to the scattering effect from the light‐induced O^δ‐^ ions. The photoresponsivity and the specific detectivity are found to be >10^4^ AW^‐1^ and >10^13^ Jones, respectively, which are significantly higher than those in standard MoTe_2_ devices. Moreover, through tuning the light programming time, the photodetection behavior of the MoTe_2_/a‐STO heterostructure experiences a dynamic evolution from negative to positive. This is due to the optically controllable modulation of the interfacial states, which is further confirmed by the X‐ray photoelectron spectroscopy and photoluminescence measurements. It is envisioned that the 2D material/a‐STO heterostructure could be a potential platform for exploring new functional devices.

## Graphical abstract

Anomalous negative photoresponse is identified in the pristine molybdenum ditelluride (MoTe_2_)/amorphous strontium titanium oxide (a‐STO) heterostructure with remarkable photoresponsivity and detectivity. Through tuning the light programming time, its photodetection behavior experiences a dynamic evolution from negative to positive due to the optically controllable modulation of the interfacial states. These results envision the 2D material/a‐STO heterostructure as a potential platform for exploring new heterogeneous integrated optoelectronics.

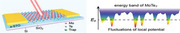

Heterostructures, formed by artificially stacking dissimilar materials, often lead to new emerging properties different than their individual constituents.^[^ ^1^, ^2^, ^3^ ^]^ Conventional heterostructures, namely, the heterostructures realized from the same crystal structural family, have been a common approach due to their advantages in maintaining the material integrity and minimizing the structural defects.^[^ ^4^, ^5^ ^]^ Recent efforts, however, have been devoted to the development of heterostructures combining a variety of material families, such as traditional compound semiconductors,^[^ ^6^, ^7^ ^]^ perovskite oxide,^[^ ^8^ ^]^ and more recently 2D atomic layered materials due to their potential for multifunctional devices.^[^ ^9^ ^]^ The 2D layered materials have established themselves as a central focus in the last decade, owing to their extraordinary structural and physical properties. The behavior of 2D materials is mostly determined by the surface/interface quality and properties; therefore, altering their interfacial coupling by utilizing other material groups could offer great potential in achieving novel phenomena that are both scientifically and technologically relevant.

Complex transition metal oxides (TMO), exhibiting exotic electrical and magnetic properties either in the crystalline or amorphous phase, have been considered as a promising candidate for the nonconventional heterostructure with 2D materials.^[^ ^10^ ^]^ Strontium titanate (SrTiO_3_, STO), one of the typical TMO materials, has been integrated with 2D materials as a gate dielectric in the field‐effect‐transistor (FET) geometry, giving rise to a range of extraordinary phenomena.^[^ ^11^, ^12^ ^]^ The correlation between graphene and STO has been extensively investigated, revealing virtually noninteracting Dirac electrons in graphene by screening the long‐range electron–electron interactions and the potential fluctuations.^[^ ^13^ ^]^ Programmable photoconductivity was observed in black phosphorus (BP)/STO structure,^[^ ^11^ ^]^ while persistent optical gating was demonstrated in STO‐supported topological insulator.^[^ ^14^ ^]^

However, since the direct deposition of high‐quality crystalline STO (c‐STO) thin film on silicon remains challenging, most of the investigations on 2D material/STO heterostructures are restricted in the commercial bulk STO substrate, which limits the potential of integrating such heterostructure with the CMOS platform. On the other hand, amorphous STO (a‐STO), which demonstrates equally extraordinary properties as its crystalline counterpart, can be conveniently grown on complementary metal–oxide–semiconductor (CMOS) compatible dielectrics (for example, SiO_2_) using pulsed laser deposition (PLD). Therefore, 2D material/a‐STO heterostructure could offer an alternative route for practical integration in heterogeneous electronic circuitry. Exploring and understanding the behavior of such heterostructure could thus open up a new avenue for designing new electronic and optoelectronic architectures.

In this work, we report a heterostructure consisting of a bilayer of molybdenum ditelluride (MoTe_2_) and a PLD grown a‐STO thin film on a Si/SiO_2_ substrate. Structural defects on the a‐STO surface cause significant modulation of the optical/electrical properties of the MoTe_2_ through the formation of interfacial states. The MoTe_2_/a‐STO heterostructure can function as a high‐performance photodetector, in which anomalous negative photodetection behavior is observed under light illumination with different wavelengths. Such photodetector exhibits remarkable enhancements in both the photoresponsivity (>10^4^ A W^−1^) and the specific detectivity (>10^13^ Jones). Furthermore, we demonstrate that the interfacial states evolve in an optically controllable manner, which is represented by the light illumination induced modification in both the transport property and the photodetection behavior of the MoTe_2_ device. Such optically tunable interfacial states evolution can also be generalized to other 2D materials, which is validated in the heterostructures of molybdenum disulfide (MoS_2_)/a‐STO, tungsten disulfide (WS_2_)/a‐STO, tungsten diselenide (WSe_2_)/a‐STO, and molybdenum diselenide (MoSe_2_)/a‐STO.

**Figure** **1**a depicts a schematic of the MoTe_2_/a‐STO heterostructure fabricated in a two‐terminal field‐effect‐transistor configuration. An a‐STO thin film with a thickness of ≈15 nm is directly deposited on a commercial SiO_2_/Si substrate using a standard PLD method (see the Experimental Section for details). In contrast to the bulk crystalline STO, the a‐STO film exhibits a corrugated surface morphology with a plateau‐like feature as shown in Figure 1b, indicating the existence of significant amounts of structural defects. Such structural defects are considered as an effective tailoring factor at the MoTe_2_/a‐STO interface, introducing interfacial states that locally modulate the energy band structure of MoTe_2_, which is represented by the local potential fluctuations (Figure 1a).^[^ ^15^ ^]^ We also conduct contact angle measurement to qualitatively visualize the interfacial states. As shown in Figure S1 (Supporting Information), the contact angle of the a‐STO surface is 46.5°, which is effectively smaller than that of the SiO_2_ substrate (63°), suggesting a more hydrophilic surface property caused by the tailoring states. The significant modification of the interfacial properties could induce considerable variation in both the electrical and optoelectrical behavior of the MoTe_2_ device.^[^ ^15^, ^16^, ^17^ ^]^

**Figure 1:**
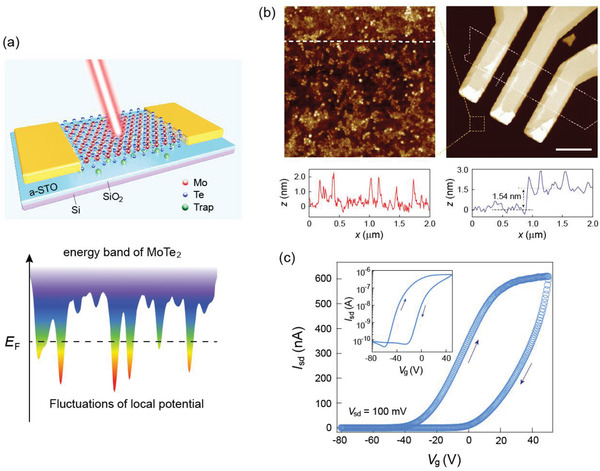
a) Schematic illustrations of the MoTe_2_/a‐STO device and the simplified energy band structure of MoTe_2_. The fluctuations of local potential are induced by the interfacial states in the heterostructure. b) AFM images of the as‐fabricated device and the enlarged area on the a‐STO thin film. Scale bar is 5 µm. c) Transport characterization of the MoTe_2_/a‐STO device under bias *V*_sd_ = 100 mV. Significant hysteresis is observed, suggesting the existence of interfacial states.

The optical image of the as‐fabricated device is shown in Figure S2a (Supporting Information), with the 2D MoTe_2_ crystal confirmed by Raman spectroscopy (Figure S2c, Supporting Information). The thickness of the MoTe_2_ flake is revealed by the atomic force microscopy (AFM) line profile as ≈1.54 nm, indicating its bilayer nature (Figure 1b). The corrugations induced by the a‐STO film can also be viewed in the 3D AFM image (Figure S2b, Supporting Information). Note that considerable surface corrugations are observed even in the MoTe_2_ covered area, indicating that the MoTe_2_ flake effectively follows the a‐STO surface morphology without semisuspension on the spikes. The basic transport characterization of the heterostructure is demonstrated in Figure 1c, displaying a typical n‐type dominated behavior. An enhanced hysteresis loop is observed in the transfer curve as compared with the MoTe_2_ device fabricated on standard SiO_2_ substrate (Figure S3a, Supporting Information), which further confirms the effect of the interfacial states. Nevertheless, as shown in Figure S4a (Supporting Information), the output characterization at multiple gate voltages still exhibits nearly ideal linear relationship, illustrating the negligible influence of the interfacial states on the quality of the electrical contacts.

Since the photodetection behavior of the 2D materials has been proved to be sensitive to the interfacial states,^[^ ^18^, ^19^ ^]^ we utilize the MoTe_2_/a‐STO heterostructure as a photodetector to further explore its properties. We first characterize the bare a‐STO substrate to exclude the possibility of its intrinsic photoresponse (Figure S5, Supporting Information). **Figure** **2**a shows the output curves of the heterostructure photodetector under both dark and illumination (*λ* = 638 nm) conditions at a backgate voltage of *V*_g_ = 0 V. A lower channel current under illumination is observed through the entire window of applied source–drain bias *V*_sd_, indicating a negative photodetection behavior. The photocurrent *I*_ph_ at each *V*_sd_ is extracted using *I*_ph_ = *I*_light_ – *I*_dark_, where *I*_light_ and *I*_dark_ are the source–drain currents under light illumination and dark condition, respectively, and a linear dependence on the *V*_sd_ is demonstrated (Figure S4b, Supporting Information). To further illustrate the photodetection property of the MoTe_2_/a‐STO photodetector, we investigate the *I*_ph_ at various *V*_g_ and light illumination power (Figure 2b,c). The negative *I*_ph_ is consistently observed at each *V*_g_ under different illumination power, suggesting a universal photodetection characteristic in the pristine MoTe_2_/a‐STO heterostructure. Note that the saturated *I*_ph_ at higher *V*_g_ is due to the saturation of the transport current as shown in the transfer characterization (Figure 1c). The stability of such a negative photoresponse is also investigated, as shown in Figure S6 (Supporting Information). We note that previous works, in general, reveal a positive *I*_ph_ in the MoTe_2_ phototransistors fabricated on SiO_2_ substrate, which originates from the introduction of photocarriers in MoTe_2_ under illumination.^[^ ^20^, ^21^ ^]^ This is distinct from the photoresponse behavior in our MoTe_2_/a‐STO heterostructure transistor, in which negative photocurrent is identified at all the biases and gates. Such negative photoresponse behavior is also in contrast to that in a system dominated by the interfacial states, where positive photodetection with relatively long response time is expected due to the charge trapping induced photogating effect.^[^ ^22^, ^23^ ^]^

**Figure 2:**
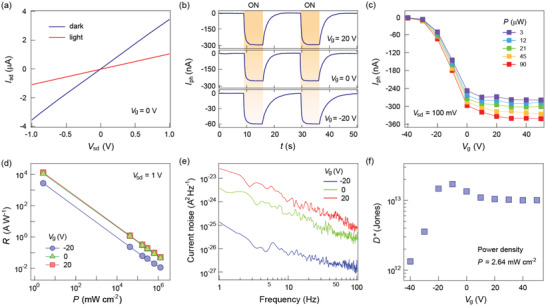
a) *I*_sd_–*V*_sd_ characterization of the MoTe_2_/a‐STO device under both dark and illuminated conditions at *V*_g_ = 0 V. b) Time‐dependent photocurrent *I*_ph_ of the device at *V*_g_ = 20, 0, and −20 V. Bias *V*_sd_ = 100 mV. c) Photocurrent versus gate voltage under different light power. The device shows a negative photocurrent within the entire experimental window. d) Power‐dependent photoresponsivity of the device at *V*_g_ = 20, 0, and −20 V. Bias *V*_sd_ = 1 V. The photoresponsivity reaches up to 10^4^ A W^−1^ at low power density. Note that the light with high power density is from a focused laser beam, while the light with low power density is from an unfocused xenon spot. e) Current noise spectra of the device at *V*_g_ = 20, 0, and −20 V. f) Gate‐dependent specific detectivity calculated at lowest power density, with the highest value exceeding 10^13^ Jones.

Moreover, the photocurrent generation in the MoTe_2_/a‐STO device is significantly improved compared with the SiO_2_ supported MoTe_2_ photodetector measured in the same conditions. To quantitatively illustrate the performance of the MoTe_2_/a‐STO photodetector, we determine the photoresponsivity as *R* = *I*_ph_ / (*P*·*S*), where *P* is the light intensity, and *S* is the effective area under illumination. As shown in Figure 2d, the photoresponsivity of the MoTe_2_/a‐STO photodetector reaches up to 10^4^ A W^−1^ as decreasing light power density, demonstrating more than two orders of magnitude enhancement on the basis of a standard MoTe_2_/SiO_2_ device (Figure S3d, Supporting Information). The current noise spectra at multiple *V*_g_ are depicted in Figure 2e, the noise equivalent power (NEP) is then calculated as NEP = noise density/*R*. The detailed information about how to measure the current noise spectra of the MoTe_2_/a‐STO heterostructure is demonstrated in Figure S7 (Supporting Information). The NEP saturates at ≈2×10^−5^ pW Hz^−1/2^ with increasing *V*_g_, suggesting the great potential of applying MoTe_2_/a‐STO heterostructure in ultra‐low light power detection (Figure S8, Supporting Information). We also investigate the gate‐dependent behavior of the specific detectivity defined as *D*: = (*B*·*S*)^1/2^/NEP, where *B* is the measuring bandwidth. The value of *D*: peaked at *V*_g_ = −10 V, followed by a gradual saturation at ≈10^13^ Jones (Figure 2f), which is considerably higher than those reported in other 2D materials.^[^ ^24^, ^25^, ^26^ ^]^ Additionally, the photodetection behavior of the MoTe_2_/a‐STO heterostructure at other wavelengths (*λ* = 515, 473, and 405 nm) is demonstrated in Figure S9 (Supporting Information), in which negative photoresponse and remarkably enhanced photodetection performance are consistently observed. The wider spectrum photoresponse of the heterostructure is also investigated (Figure S10, Supporting Information), where the *R* gradually decreases with increasing wavelength. Nevertheless, the *R* can still be maintained in the magnitude of ≈10^3^ A W^−1^, indicating the potential of using such heterostructure for broadband photodetector.

To obtain comprehensive information on the photoresponse behavior of the MoTe_2_/a‐STO heterostructure, we dynamically monitor the device performance under several consecutive light illumination periods with the duration fixed at 75 s, and the gate voltage *V*_g_ is set to be negative within each illumination process. **Figure** **3**a shows the time‐dependent photocurrent of the MoTe_2_/a‐STO device. The negative photocurrent with decreasing magnitude is persistently observed until the total illumination time *t*_pro_ = 450 s. However, when the *t*_pro_ reaches 600 s, the polarity of the photocurrent switches to positive. Moreover, the evolution of the photocurrent is accompanied by the shortening of the photoresponse time, which finally approaches the intrinsic fast photoresponse speed of a standard MoTe_2_ photodetector. In addition to the photoresponse behavior, the transport characterization after each illumination period was also investigated (Figure 3b), which exhibits a significant electron doping effect with the weakened hysteresis as increasing the illumination time. The evolution of the transport characterization suggests a photogating effect that has been previously observed in the 2D photodetectors with the assistance of interfacial states.^[^ ^27^, ^28^ ^]^ During the illumination process, the photogenerated holes are continuously trapped by the interfacial states, a process that maintains the electron concentration in the MoTe_2_ channel while screening the interfacial scattering centers. Therefore, in the final state after the sufficiently longtime illumination, the MoTe_2_ channel is heavily n‐doped with an effective screening of the external perturbations, which makes its photoresponse behavior approach that of the standard MoTe_2_ phototransistor. The summaries of the photocurrent and response time evolution are demonstrated in Figure 3c,d, respectively. The photocurrent progressively evolves from −160 to 28 nA, with two orders of magnitude drop in the response time, further confirming that the photoresponse of the device approaches its intrinsic characteristics after the prolonged light illumination. Note that the bare a‐STO substrate after illumination shows no difference compared with its pristine counterpart (Figure S11, Supporting Information).

**Figure 3:**
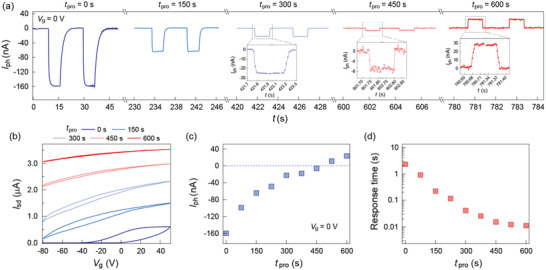
a) Time‐dependent photocurrent at *V*_g_ = 0 V as prolonging the light programming time *t*_pro_. Wavelength *λ* = 638 nm, bias *V*_sd_ = 100 mV. The photocurrent polarity switches from negative to positive after the prolonged light illumination, with the observation of decreased photoresponse time. b) Illumination‐dependent transport characterization of the heterostructure. The device shows a significant n‐type doping effect with reduced hysteresis after light programming. Evolution of the c) photocurrent and d) response time as a function of the programming time *t*_pro_.

Additionally, the light‐controlled evolution of the photoresponse behavior under other gate voltages are investigated, where the photocurrent possesses a consistent polarity switching as increasing the illumination time (Figure S12a, Supporting Information). Such polarity switching is also observed at other wavelengths (Figure S13a, Supporting Information). Note that the photoresponse time depends only on the illumination process, with little effects from both the gate voltage and the wavelength (Figures S12b and S13b, Supporting Information). We also investigated the a‐STO with different thicknesses from 15 to 50 nm, incorporating with MoTe_2_ from 1.5 nm to around 10 nm (Figures S14 and S15, Supporting Information). The electrical transport and evolution of photoresponse are consistent for the a‐STO substrates and the MoTe_2_ samples with various thicknesses. These results indicate that the evolution behavior of the MoTe_2_/a‐STO heterostructure is largely determined by the conditions of the interfacial states, which can be effectively modulated by controlling the light illumination procedure.

Although the photodetection evolution in the MoTe_2_/a‐STO heterostructure can be described by the light‐induced changes of the interfacial states, understanding its initial anomalous negative photoresponse is essential to explore the real application of such heterostructure. It has been demonstrated in the previous work that the as‐deposited STO thin film could release oxygen atoms under light illumination.^[^ ^29^, ^30^ ^]^ Moreover, the oxygen vacancies in PLD‐grown STO film could introduce optically‐active gap states that provide photogenerated electrons, particularly in the case of amorphous STO thin film.^[^ ^31^, ^32^ ^]^ These released oxygen atoms can then trap the photoexcited electrons to form negatively charged oxygen ions O*^*δ*^* ^−^, where *δ* is the effective charge on each oxygen atom.^[^ ^30^ ^]^ We, therefore, propose a model to synergistically describe the initial and evolved photodetection behavior in the MoTe_2_/a‐STO heterostructure, as shown in **Figure** **4**a. Initially, the O*^*δ*^* ^−^ ions created by the light illumination are able to migrate within the a‐STO film or move toward the MoTe_2_/a‐STO interface. These O*^*δ*^* ^−^ ions behave as the scattering centers to degrade the MoTe_2_ channel current, which is represented by the negative photocurrent. When the light is off, the photogenerated electrons recombine with the charged gap states, and the O*^*δ*^* ^−^ ions revert to oxygen atoms that are reabsorbed into the a‐STO film, the channel current is thus restored due to the disappearance of the scattering centers. Second, during the light illumination process, while applying negative *V*_g_, the photogenerated holes in MoTe_2_ are continuously trapped at the interfacial states under the external electric field, leading to the electron accumulation and strong n‐type doping effect in the MoTe_2_ channel. The band alignment diagram is shown in Figure S16 (Supporting Information) to explain the hole trapping and transporting in the light programming process. These trapped holes can also screen the scattering effect arising from the O*^*δ*^* ^−^ ions, thus accelerating the charge separation/recombination, which results in the reduction of negative photocurrent and photoresponse time while increasing the light programming time. Similarly, in the final stage after a sufficiently longtime illumination, significant amounts of holes are trapped at the interface and completely screen the O*^*δ*^* ^−^ scattering centers. As a consequence, the photodetection behavior of the MoTe_2_/a‐STO heterostructure approaches that of a heavily n‐doped standard MoTe_2_ device, where positive photocurrent with a fast response speed is expected. It is of note that the positive photocurrent will gradually decrease, and eventually evolves to the negative polarity when the retention time is 8 h (Figure S17, Supporting Information), suggesting the gradual release of the trapped holes. Upon prolonging the time, the amount of trapped holes progressively decreases, which is not sufficient to screen the scattering effect from the O*^*δ*^* ^−^ ions, leading to the change of the polarity from positive to negative. Additionally, applying an opposite gate could accelerate the release of the trapped holes, which reverses the photocurrent polarity in a shorter time (Figure S18, Supporting Information).

**Figure 4:**
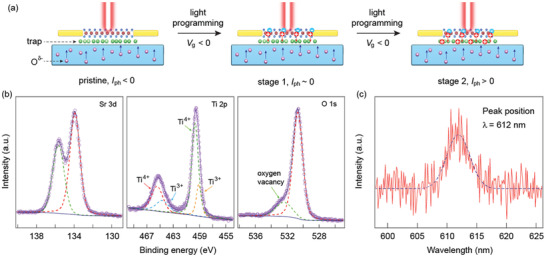
a) Schematic illustration of the evolution of the interfacial state of the MoTe_2_/a‐STO device. The green spheres indicate the traps at the interface, while the pink ones are the charged oxygen atoms. The red and blue circles represent the photogenerated hole and electron, respectively. In the pristine device, the light‐induced O*^*δ*^* ^−^ scattering centers lead to the initial negative photocurrent. After light programming under *V*_g_ < 0, the photogenerated holes in MoTe_2_ are continuously trapped at the interfacial states, which screens the scattering effect arising from the O*^*δ*^* ^−^ ions. In the intermediate stage 1, the negative photocurrent caused by scattering is balanced with the intrinsic positive photocurrent, therefore, no photocurrent is detected in the device. A positive photocurrent is finally observed when the O*^*δ*^* ^−^ ions are completely screened by the trapped holes, as shown in stage 2. b) XPS spectra of the Sr 3d, Ti 2p, and O 1s core levels in the a‐STO thin film. The emergence of Ti^3+^ peaks indicates the existence of the oxygen vacancies, which is further confirmed by the O 1s core level. c) PL spectrum of the a‐STO thin film. The peak located at 612 nm demonstrates the oxygen vacancy induced gap states, which is consistent with the XPS spectra.

To validate the existence of oxygen vacancies in the a‐STO film, we perform the X‐ray photoelectron spectroscopy (XPS) measurement on the as‐deposited a‐STO thin film, with all the spectra fitted with Gaussian/Lorentzian mixed functions (Figure 4b). The locations of the two Sr 3d core level peaks are consistent with previous reports, indicating that the oxidation state of Sr is insensitive to the phase of the STO film.^[^ ^33^, ^34^ ^]^ However, the Ti 2p spectrum demonstrates two additional peaks on the basis of the Ti^4+^ oxidation state, suggesting the appearance of the Ti^3+^ oxidation state due to the emergence of the oxygen vacancies.^[^ ^35^, ^36^ ^]^ The corresponding O 1s spectrum also reveals an extra peak located at the higher binding energy, further confirming the significant amounts of oxygen vacancies in the a‐STO film.^[^ ^37^ ^]^ On the other hand, to illustrate the oxygen vacancy induced gap states, we investigate the photoluminescence (PL) spectrum of the a‐STO film at room temperature (Figure 4c). A PL peak centered at wavelength *λ* = 612 nm is observed, which is absent in a commercial STO substrate, indicating the existence of gap states. Note that the PL peak position is consistent with the red illumination band caused by oxygen vacancies in STO film.^[^ ^38^ ^]^ These results validate our model for describing the photodetection behavior of the MoTe_2_/a‐STO heterostructure.

2D materials beyond MoTe_2_ share a similar layered structure with varied electronic and optoelectronic properties. To substantially explore the potential of 2D material/a‐STO heterostructure, we investigate MoS_2_/a‐STO, WS_2_/a‐STO, WSe_2_/a‐STO, and MoSe_2_/a‐STO heterostructures, respectively. The optical image of the as‐fabricated MoS_2_/a‐STO heterostructure transistor is shown in Figure S19a (Supporting Information). The Raman spectrum of the MoS_2_ flake reveals two characterization peaks with a separation of 21.5 cm^−1^, indicating the bilayer nature of the flake (Figure S19b, Supporting Information). We observe similar photodetection behavior in the pristine MoS_2_/a‐STO device as compared with that of the MoTe_2_/a‐STO heterostructure, as illustrated in **Figure** **5**a,b. Moreover, the transport and photoresponse properties of the MoS_2_/a‐STO device demonstrate consistent evolution characteristics that are controlled by the light illumination process, indicating the existence of the progressively evolved interfacial states (Figure 5c,d). Additionally, the WS_2_/a‐STO, WSe_2_/a‐STO, and MoSe_2_/a‐STO heterostructures also exhibit photoresponse behavior that is similar to both the MoTe_2_ and MoS_2_ (Figures S20–S22, Supporting Information), implying that the optically controlled evolution of interfacial states is generally applicable in 2D material/a‐STO heterostructures. On the other hand, the photoresponsivity of these three photodetectors are determined as 2.9 × 10^4^ A W^−1^ (MoS_2_), 4.3 × 10^3^ A W^−1^ (WS_2_), 5.9 × 10^3^ A W^−1^ (WSe_2_), and 3.1 × 10^4^ A W^−1^ (MoSe_2_), respectively, promising the 2D material/a‐STO heterostructure as a potential platform toward high‐performance photodetector application.

**Figure 5:**
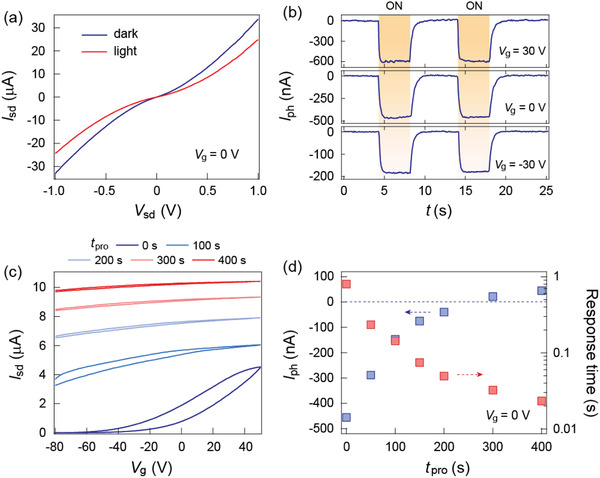
a) *I*_sd_–*V*_sd_ characterization of the MoS_2_/a‐STO device under both dark and illuminated conditions at *V*_g_ = 0 V. Wavelength *λ* = 638 nm. b) Time‐dependent photocurrent *I*_ph_ of the device at *V*_g_ = 30, 0, and −30 V. Bias *V*_sd_ = 100 mV. c) Illumination‐dependent transport characterization of the heterostructure. The device shows a significant n‐type doping effect with reduced hysteresis after light illumination, in good agreement with the MoTe_2_/a‐STO device. d) Evolution of the photocurrent and response time as a function of the programming time *t*_pro_.

In summary, we have fabricated a heterostructure based on the 2D MoTe_2_ flake and PLD grown a‐STO thin film that functions as a high‐performance photodetector and demonstrated optically controlled photodetection behavior driven by the evolution of interfacial states. The pristine device reveals anomalous negative photocurrent, where the photoresponsivity and specific detectivity reach up to ≈10^4^ A W^−1^ and ≈10^13^ Jones, respectively, which are remarkably higher than those of the MoTe_2_ device on standard SiO_2_ substrate. Furthermore, we demonstrate that the photoresponse behavior of the MoTe_2_/a‐STO heterostructure is highly tunable with light programming time, where the photocurrent polarity can be effectively modulated from negative to positive with two orders of magnitude to shorten the photoresponse time. The initial negative photocurrent originates from the scattering effect caused by the light‐induced O*^*δ*^* ^−^ ions in the a‐STO film, while the light controllable photoresponse evolution is realized by the continuously trapping of photogenerated holes from MoTe_2_ at the interfacial states. Such light‐tunable photodetection behavior can also be generalized to other 2D materials, which is confirmed in the heterostructures of MoS_2_/a‐STO, WS_2_/a‐STO, WSe_2_/a‐STO, and MoSe_2_/a‐STO. Our work promises 2D material/a‐STO architecture as a potential candidate for the integration into CMOS compatible platform, envisioning their opportunities for designing new heterogeneous integrated electronics.

## Experimental Section

##### PLD Growth of a‐STO Thin Film on SiO_2_/Si Substrate

The a‐STO thin film was deposited on SiO_2_/Si substrate by pulsed laser deposition using a KrF excimer laser (*λ* = 248 nm) with a repetition rate of 2 Hz and laser fluence of 1.3 J cm^−2^. The growth temperature and oxygen partial pressure were 640 °C and 10 mTorr, respectively. After deposition, the sample was cooled down to room temperature with a cooling rate of 10 °C min^−1^ in the deposition pressure. Commercial STO single crystal was used as target. The growth rate was calibrated with the in situ reflection high‐energy electron diffraction oscillations obtained during the growth of single‐crystalline STO thin film on TiO_2_‐terminated STO (001) substrate.

##### Device Fabrication and Characterization

The MoTe_2_, MoS_2_, WS_2_, WSe_2_, and MoSe_2_ flakes were mechanically exfoliated from their bulk crystals (HQ graphene) using a scotch tape and transferred onto the PLD grown a‐STO substrate. The exfoliated flakes were located by using a high‐resolution microscope (Nikon Eclipse LV100D) followed by the spin coat of polymethyl methacrylate (PMMA) photoresist. The transistor channel was defined using the conventional e‐beam lithography technique (EBL). After lithography, metal electrodes Ti (5 nm) and Au (80 nm) were thermally deposited on the flakes in a high vacuum chamber (≈10^−7^ mbar). The devices were then liftoff in acetone solution followed by the wire‐bond onto a LCC chip carrier. The chip carrier was then loaded in a custom designed high vacuum system (≈10^−8^ mbar) for electrical and optoelectrical measurements. The electrical and optoelectrical measurements were conducted using an Agilent 2912A source measure unit. Four laser beams (638, 515, 473, and 405 nm) and an exon light source configured with a monochromator were used to illuminate the device. The light density was calibrated by THORLABS GmbH (PM 100A) power meter. To acquire the noise spectra, the source terminal of the as‐fabricated transistor was dc‐coupled to Stanford Research SR570 low noise current preamplifier. The output of this current amplifier was recorded by an HP 35670A dynamic signal analyzer. The frequency ranges from 1 to 100 Hz for each noise spectrum measurement.

## Conflict of Interest

The authors declare no conflict of interest.

## Supporting information

Supporting Information
