# Supplementary material for: Optically Controllable 2D Material/Complex Oxide Heterointerface
Source: Adv Sci (Weinh). 2020 Aug 20;7(21):2002393. doi: 10.1002/advs.202002393 (PMC7610330; doi:10.1002/advs.202002393)
Supplement: Supplementary file 1 — Supporting Information [file ADVS-7-2002393-s001.pdf]

© 2020 Wiley-VCH GmbH

ADVANCED SCIENCE
Open Access

Supporting Information

for *Adv. Sci.,* DOI: 10.1002/advs.202002393

Optically Controllable 2D Material/Complex Oxide Heterointerface

Tao Liu, Cheng Han, Du Xiang, Kun Han, Ariando Ariando, and Wei Chen**

Supporting Information

Optically controllable 2D material/complex oxide hetero-interface

*Tao Liu, Cheng Han, Du Xiang**, *Kun Han, Ariando Ariando, Wei Chen**

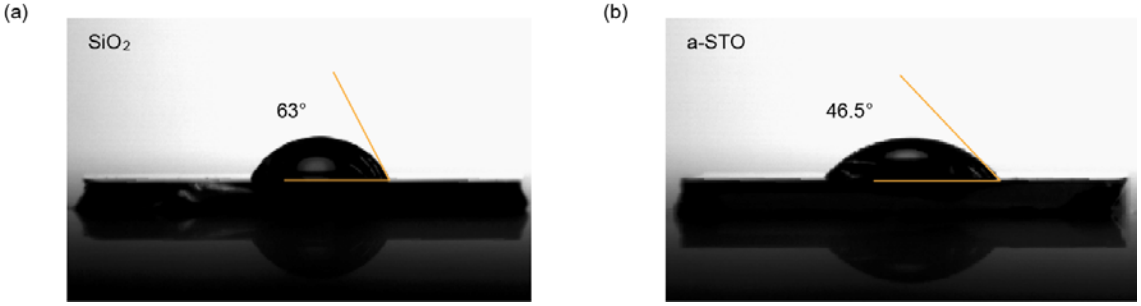

Figure S1 | Image of water contact angles. (a) SiO_2 substrate and **(b)** a-STO substrate. The contact angle of the a-STO substrate is 46.5 °, considerably smaller than that of SiO_2 substrate, indicating a more hydrophilic surface caused by the surface states. The volume of the water droplet is 2.5 μL in both cases.

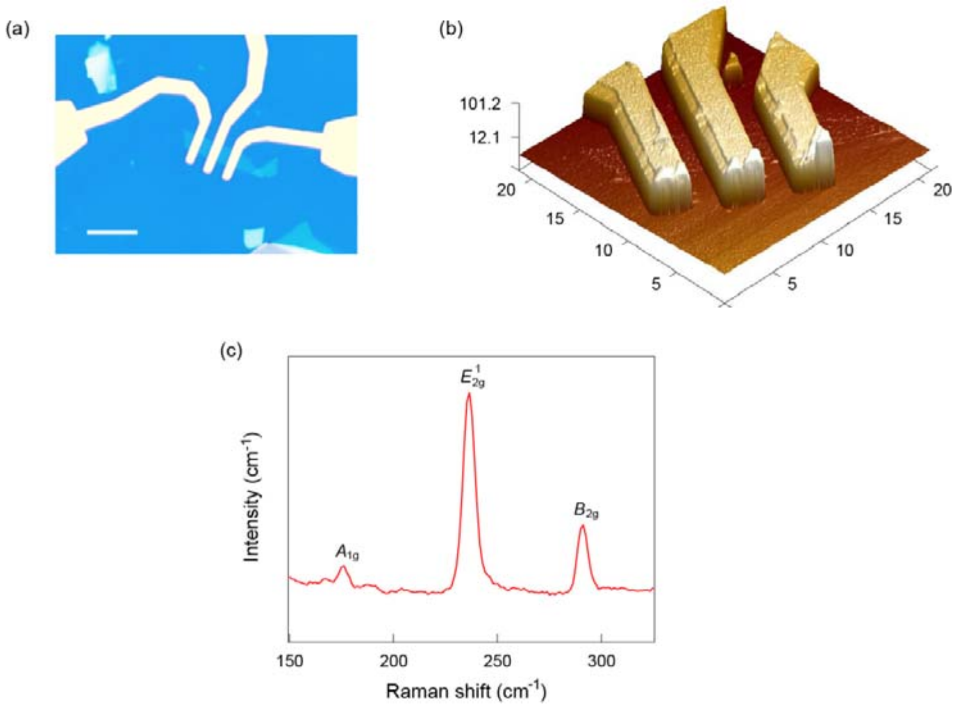

Figure S2 | Characterizations of the $MoTe_2$/a-STO heterostructure. (a) Optical and **(b)** 3D AFM image of the as-fabricated $MoTe_2$/a-STO device in a field-effect-transistor configuration. Scale bar is 10 μm. The corrugated surface morphology is revealed in the 3D AFM image. **(c)** Raman spectrum of the a-STO supported $MoTe_2$ flake. Three characteristic peaks at 172 cm^{-1} (A_{1g}), 235 cm^{-1} (E^1_{2g}), and 291 cm^{-1} (B_{2g}) are indicated.

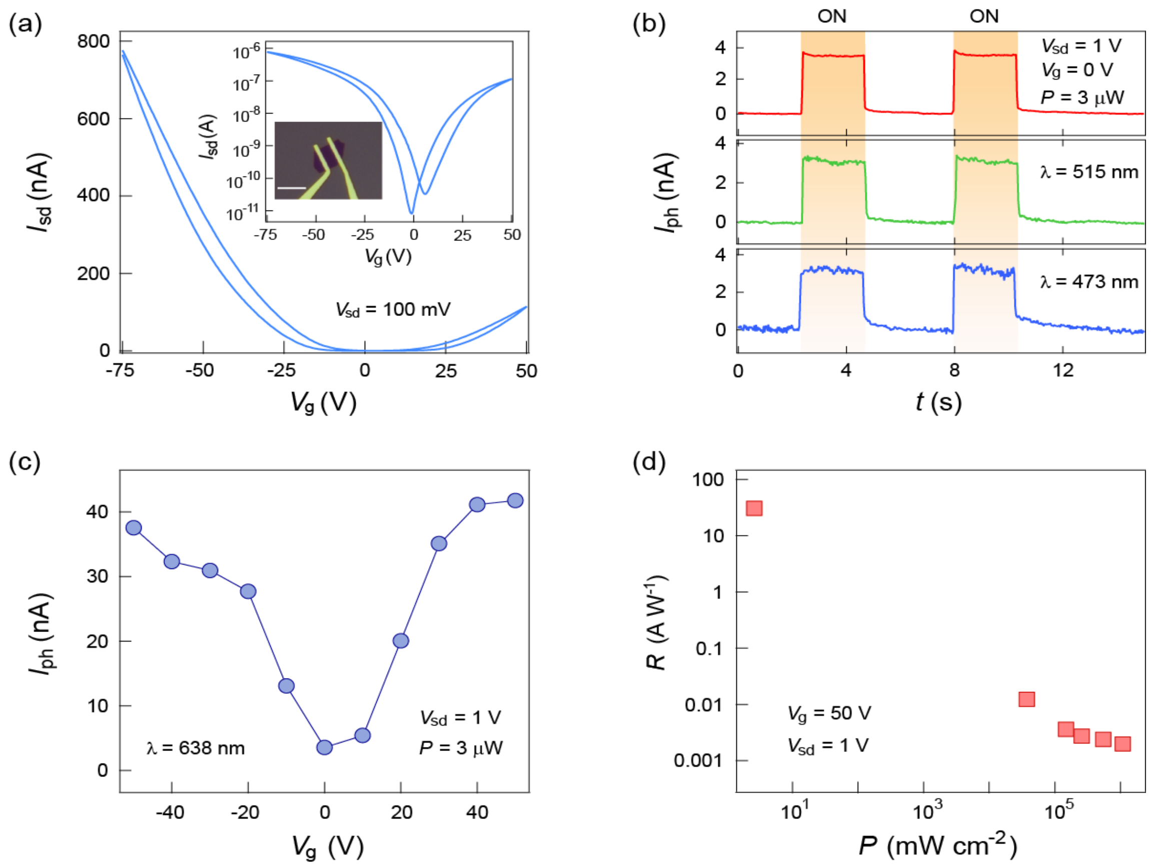

Figure S3 | Characterization of $MoTe_2$ on SiO_2 substrate. (a) Transfer curve of $MoTe_2$ on SiO_2 substrate. Weaker hysteresis is observed compared to that of a-STO supported device. Scale bar is 5 μm. **(b)** Photoresponse under the light illumination of three different wavelengths at V_g = 0 V, illustrating all-positive photocurrent. **(c)** Gate dependent photocurrent under 638 nm light illumination. Photocurrent increases as increasing the gate voltage. V_{sd} = 1 V. **(d)** The maximum photoresponsivity is extracted as ~30 A W^{-1} at V_g = 50 V. The $MoTe_2$/a-STO heterostructure demonstrates ~400 times enhancement in the photoresponsivity.

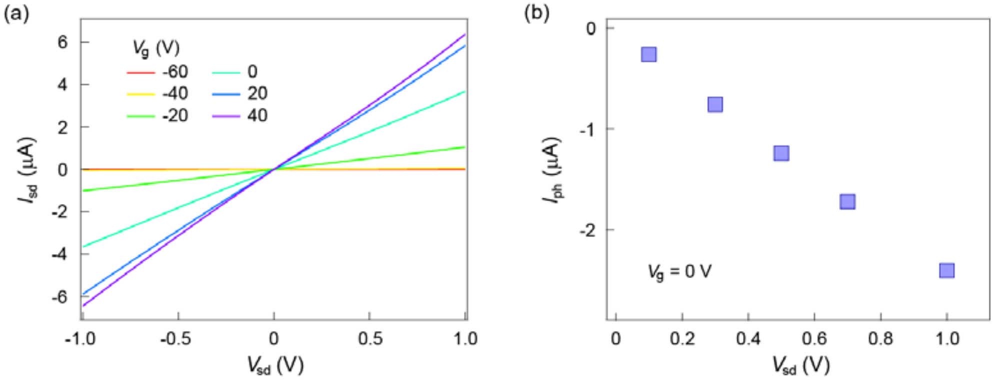

Figure S4 | I_{sd} - V_{sd} characteristics and the bias-dependent photocurrent of the same $MoTe_2$/a-STO device in the manuscript. (a) I_{sd} - V_{sd} characteristics from V_g = -60 V to 40 V, at a step of 20 V. The linear relationship between I_{sd} and V_{sd} indicates Ohmic contact of the device. **(b)** Bias-dependent photocurrent under the illumination of 638 nm light at V_g = 0 V. The photocurrent increases almost linearly with increasing V_{sd} from 0.1 V to 1 V.

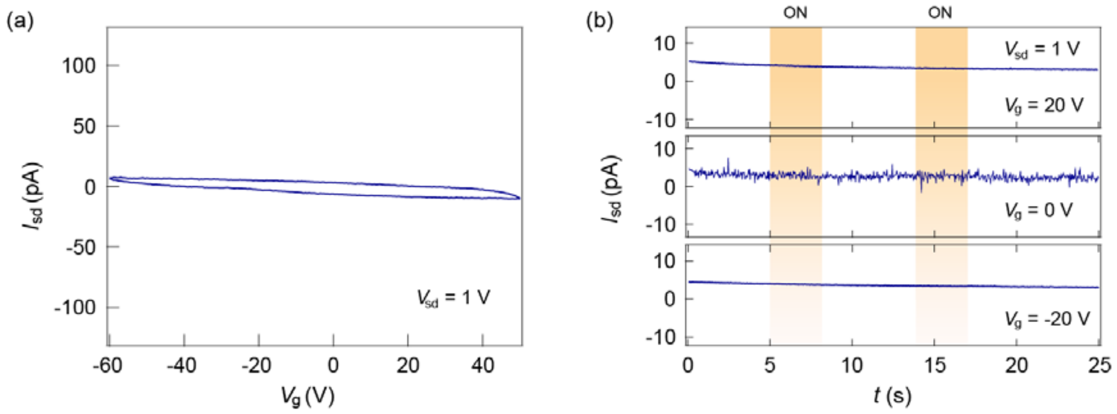

Figure S5 | Characterization of the bare a-STO substrate. (a) Transfer curve of the bare a-STO substrate, no current observed. **(b)** Photoresponse characterization of the bare a-STO substrate at V_g = 20 V, 0 V, and -20 V, no photocurrent detected.

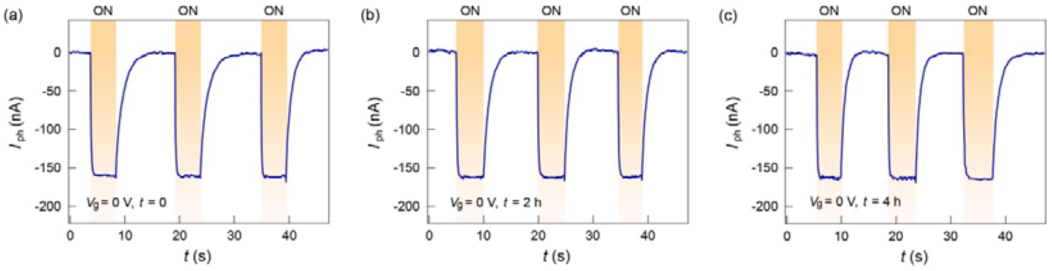

Figure S6 | Time dependent photoresponse of the pristine $MoTe_2$/a-STO heterostructure at V_g = 0 V. Here the measurement was performed at different times t = (a) 0, (b) 2 h, and (c) 4 h. The negative photocurrent is highly stable at different measurement time, and does not show any evolution under light illumination.

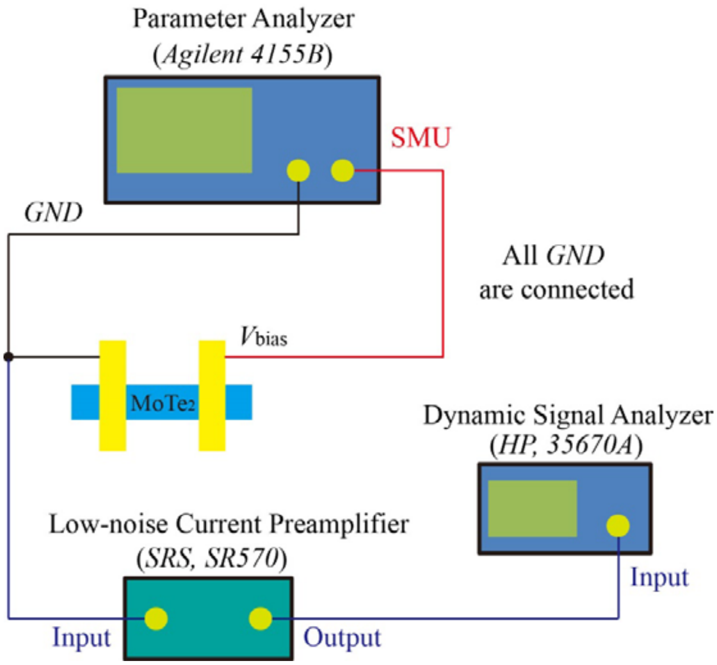

Figure S7 | Schematic of the noise measurement set-up.

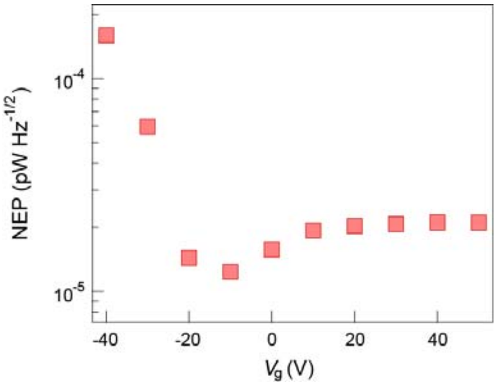

Figure S8 | Gate-dependent NEP for the calculation of specific detectivity *D shown in Figure 2(f) in the manuscript.**

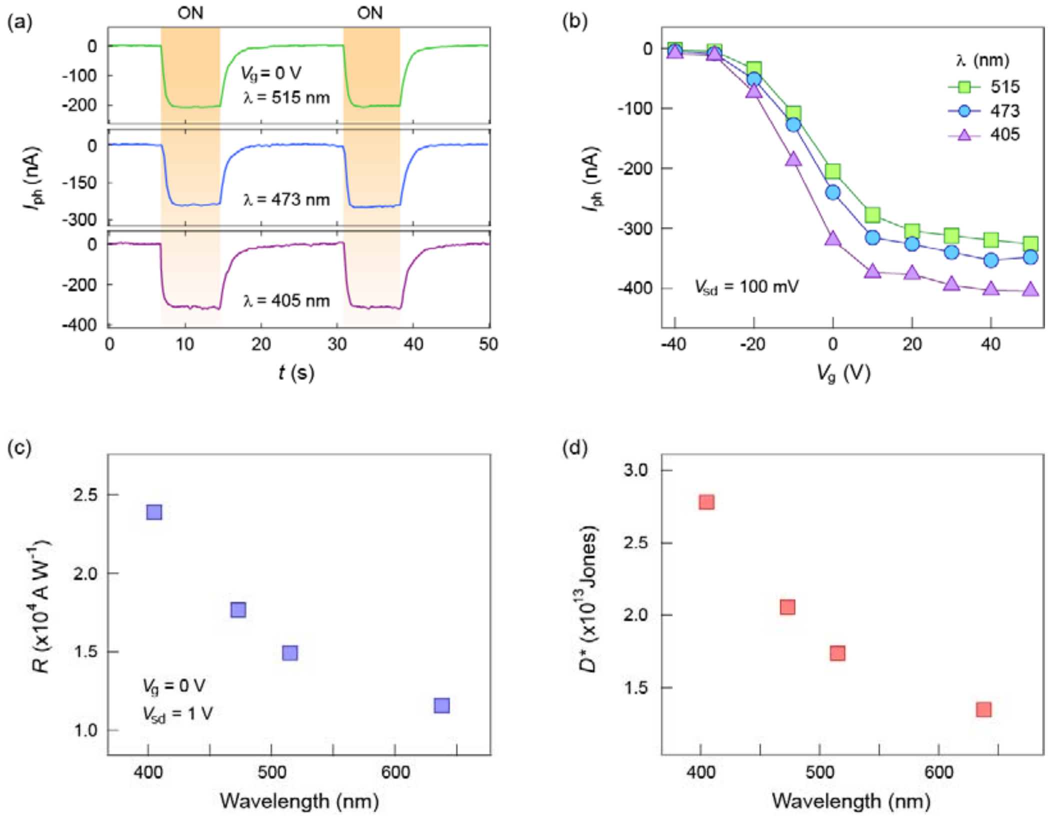

Figure S9 | Wavelength dependent photodetection behavior of the same $MoTe_2$/a-STO device in the manuscript. (a) Time-dependent photocurrent at V_g = 0 V and **(b)** Gate dependent photocurrent under the illumination of 515 nm, 473 nm, and 405 nm light, respectively. Negative photodetection behavior is consistently observed. **(c)** Photoresponsivity R and **(d)** specific detectivity D^* demonstrate the high sensitivity of the $MoTe_2$/a-STO photodetector at multiple light wavelengths.

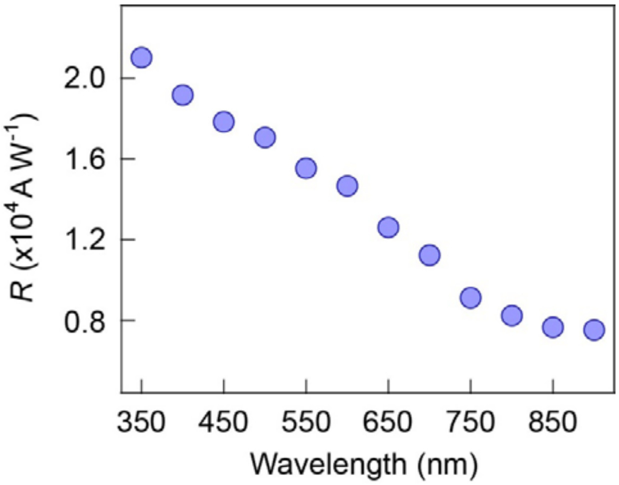

Figure S10 | Photoresponsivity as a function of wavelength from 350 nm to 900 nm.

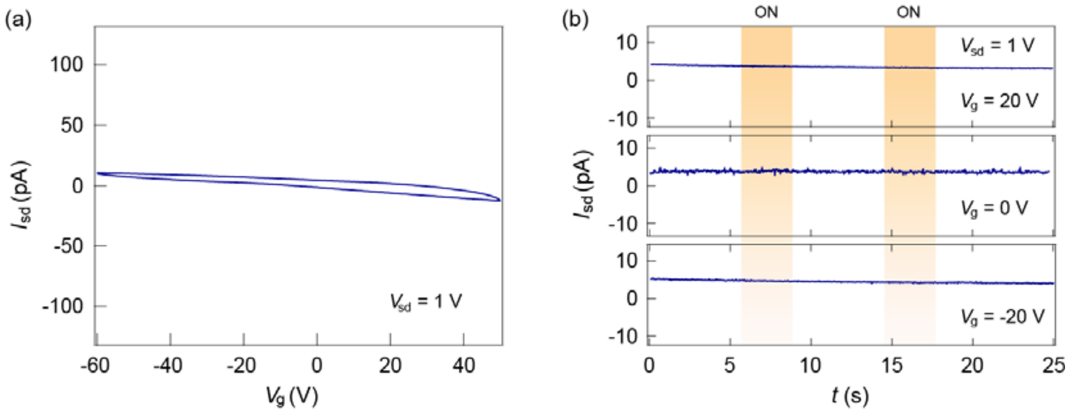

Figure S11 | Characterization of the same bare a-STO substrate as shown in Figure S5 after light illumination. (a) Transfer curve of the a-STO substrate after illumination. **(b)** Photoresponse characterization of the bare a-STO substrate after illumination. V_g = 20 V, 0 V, and -20 V. The behavior of the bare a-STO shows no difference after light illumination.

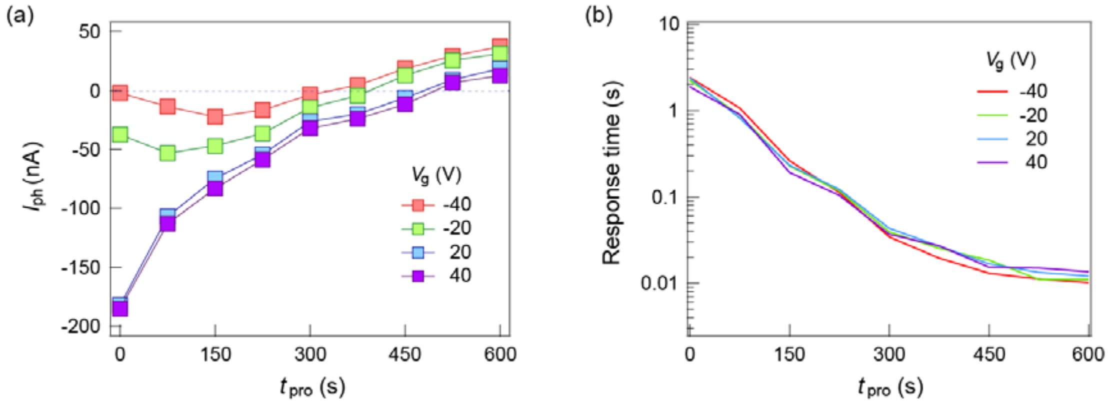

Figure S12 | Photoresponse evolution of the same $MoTe_2$/a-STO device in the manuscript at other V_g under illumination of 638 nm light. (a) Photocurrent evolution at V_g from -40 V to 40 V, at a step of 20 V. As increasing the illumination time t_{pro}, the photocurrent switches from negative to positive, which is consistent with the observation at V_g = 0 V shown in the manuscript. **(b)** Evolution of the photoresponse time. The photoresponse time demonstrates two orders of magnitude drop after illumination, which is almost independent of the gate voltage, implying that the photoresponse evolution is mainly driven by the change of interfacial states.

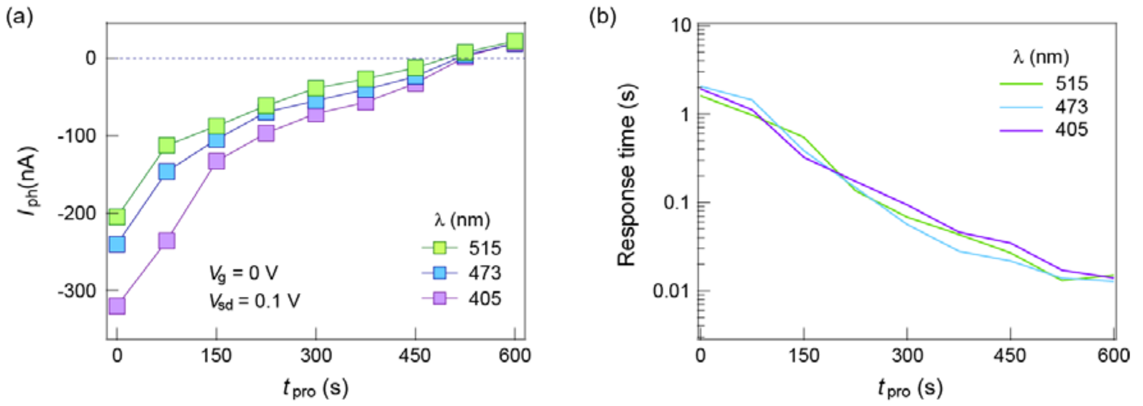

Figure S13 | Wavelength dependent photoresponse evolution of the same $MoTe_2$/a-STO device in the manuscript. (a) Photocurrent evolution at V_g = 0 V under the illumination of 515 nm, 473 nm, and 405 nm light, respectively. Negative-to-positive photocurrent switching is observed in each case. **(b)** The photoresponse time is shortened by two orders of magnitude after sufficiently long light illumination, with negligible wavelength dependence.

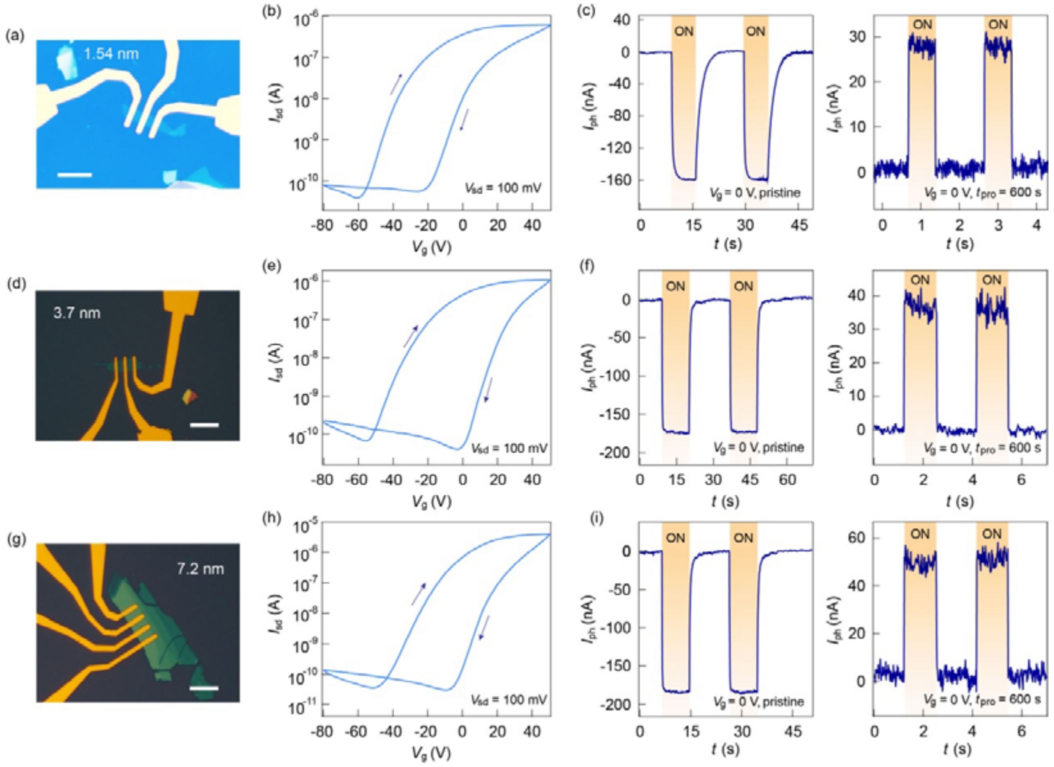

Figure S14 | $MoTe_2$ Thickness dependent photoresponse. Optical image of the $MoTe_2$/a-STO devices with different flake thicknesses (a) 1.54 nm, (d) 3.7 nm, (g) 7.2 nm. Scale bar is 5 μm. Transfer characteristics of the $MoTe_2$/a-STO devices with different thicknesses (b) 1.54 nm, (e) 3.7 nm, (h) 7.2 nm. Photoresponse of the initial and programmed $MoTe_2$/a-STO devices with different flake thicknesses (c) 1.54 nm, (f) 3.7 nm, (i) 7.2 nm.

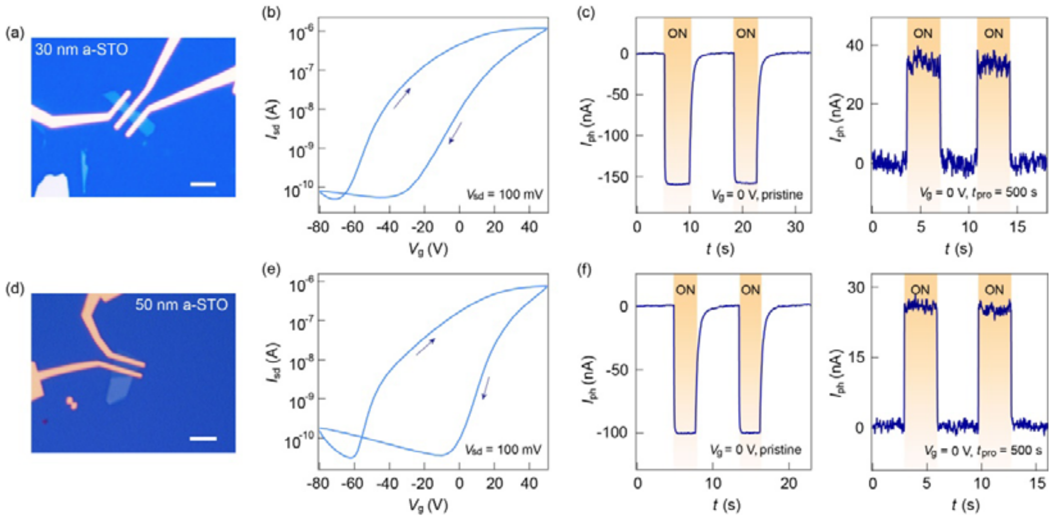

Figure S15 | a-STO Thickness dependent photoresponse. Optical image of the $MoTe_2$ devices on (a) 30 nm and (d) 50 nm a-STO substrates. Transfer characteristics of the $MoTe_2$ devices on (b) 30 nm and (e) 50 nm a-STO substrates. Photoresponse of the initial and programmed $MoTe_2$ devices on (c) 30 nm and (f) 50 nm a-STO substrates.

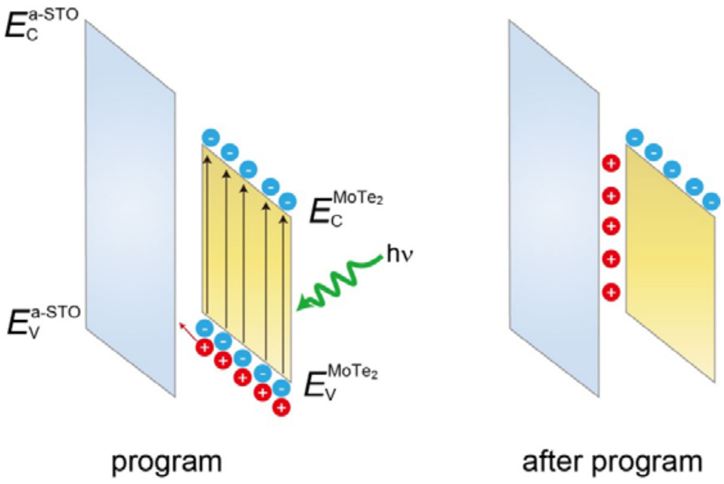

Figure S16 | Schematic of the band alignment of the $MoTe_2$/a-STO heterostructure in programming and after programming states.

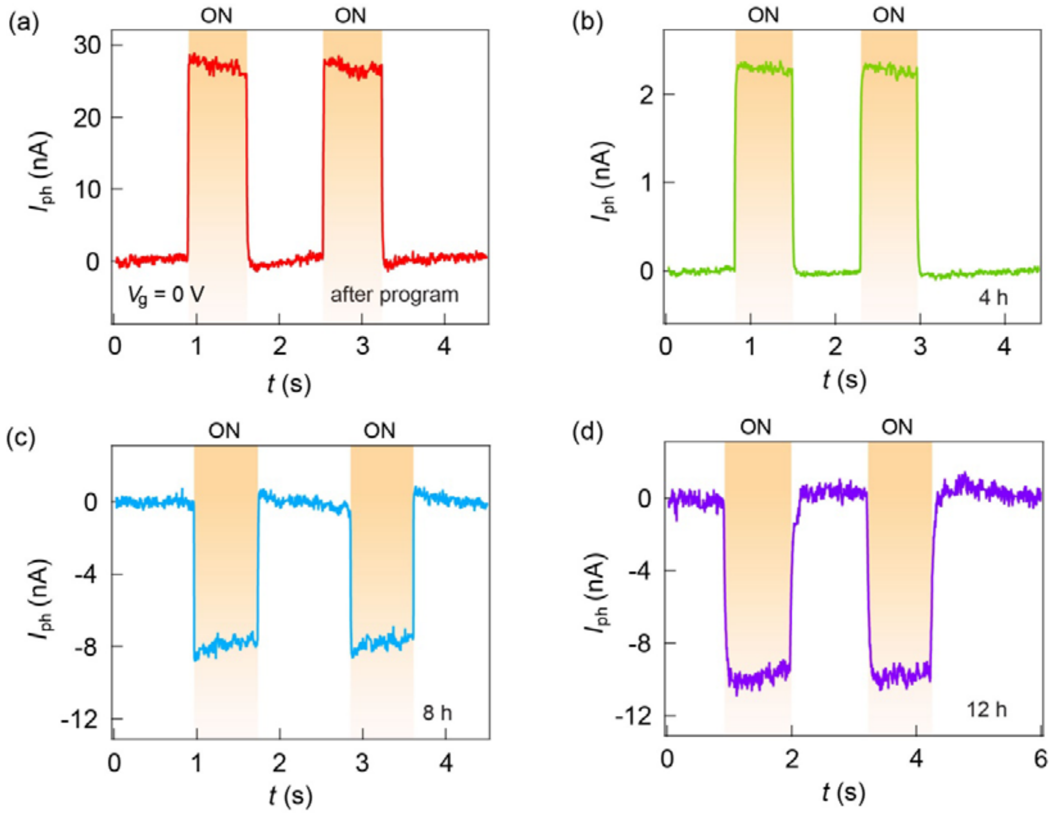

Figure S17 | Time dependent photoresponse of the programmed device as a function of retention time at (a) the beginning, (b) 4 h, (c) 8 h, and (d) 12 h.

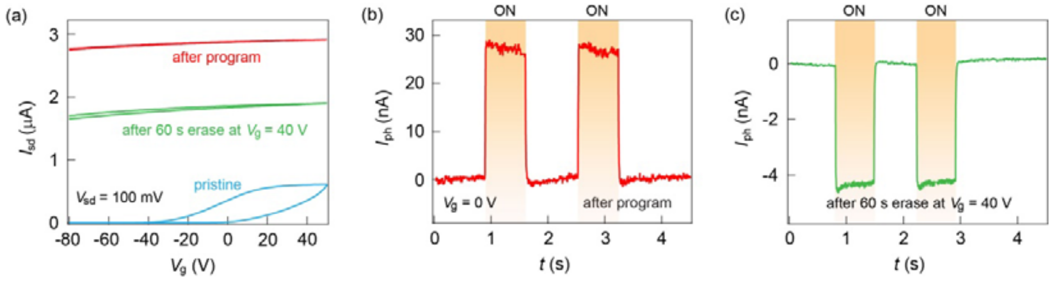

Figure S18 | Transfer and photoresponse after erasing. (a) Transfer characteristics of the pristine, programmed, and erased heterostructure device. (b) Time dependent photoresponse of the heterostructure device after light programming. (c) Time dependent photoresponse of the device after 60 s erasing at V_g = 40 V. The erasing process is defined as applying a positive gate bias on the device in dark condition.

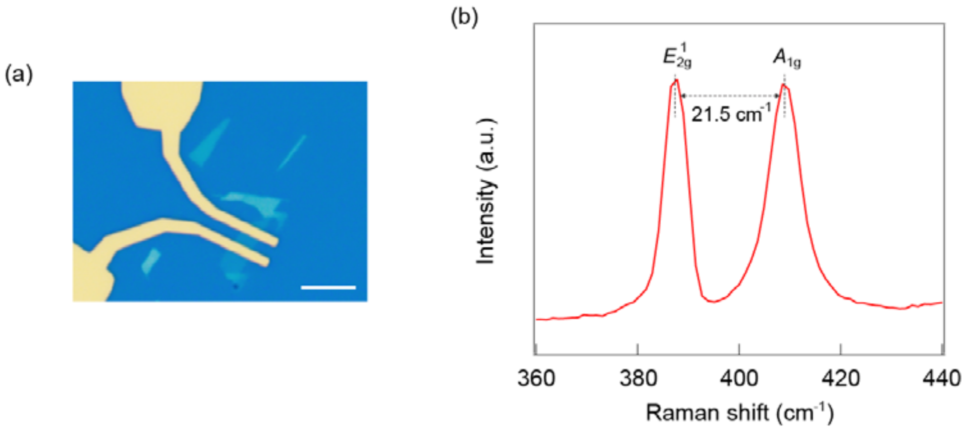

Figure S19 | Optical image and Raman characterizations of the MoS_2/a-STO heterostructure. (a) Optical image of the as-fabricated MoS_2/a-STO device in a field-effect-transistor configuration. Scale bar is 10 μm. **(b)** Raman spectrum of the a-STO supported MoS_2 flake. Two characteristic peaks at 387.5 cm^{-1} (E^1_{2g}) and 409 cm^{-1} (A_{1g}) are indicated with a separation of 21.5 cm^{-1}, illustrating its bilayer nature.

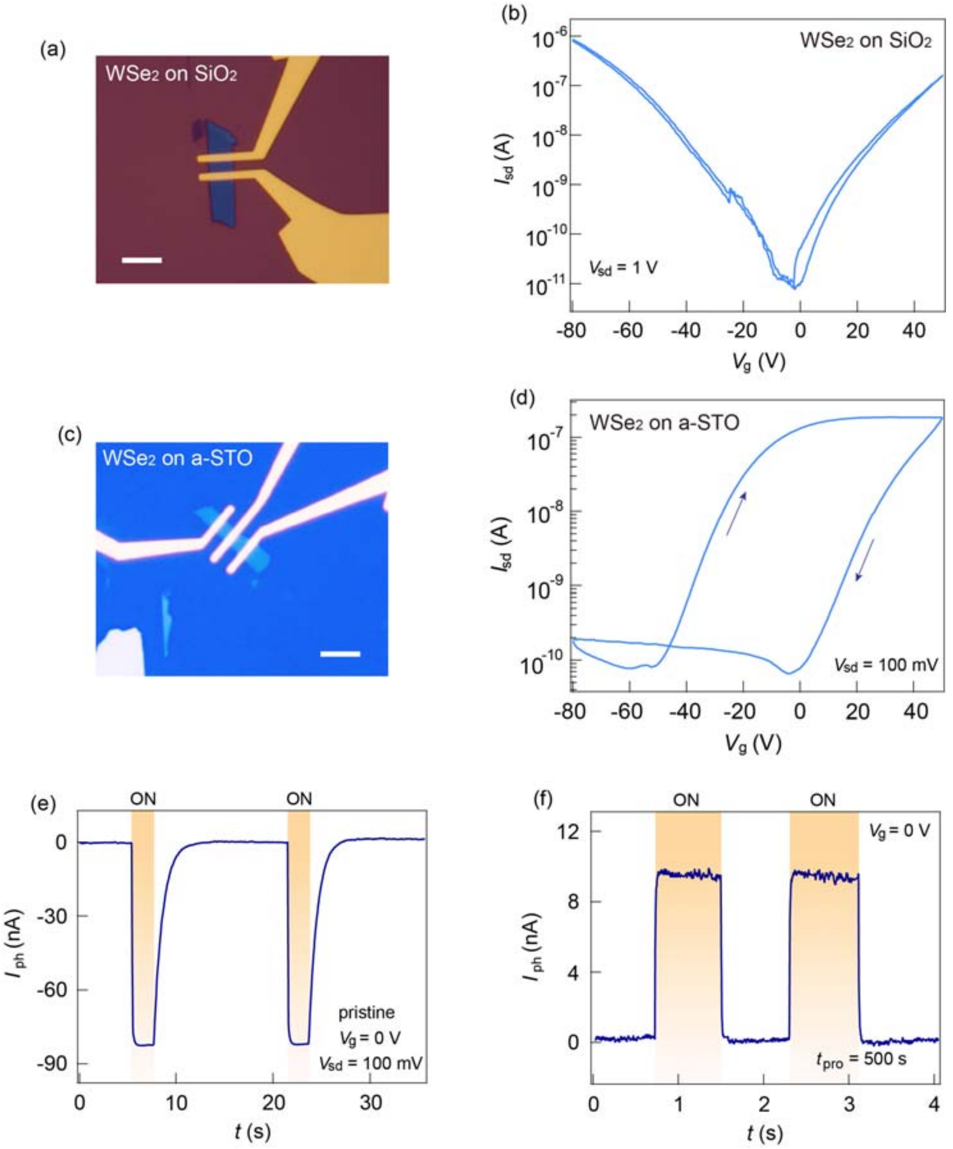

Figure S20 | WSe_2 devices on SiO_2 and a-STO substrates. Optical images of WSe_2 device on (a) standard bare SiO_2 and (c) a-STO substrates, respectively. Scale bar is 5 μm. Transfer characteristics of WSe_2 device on (b) standard bare SiO_2 and (d) a-STO substrates, respectively. Time dependent photoresponse of the WSe_2 device on a-STO substrate at the (e) pristine and (f) programmed (t_{pro} = 500 s) state, respectively.

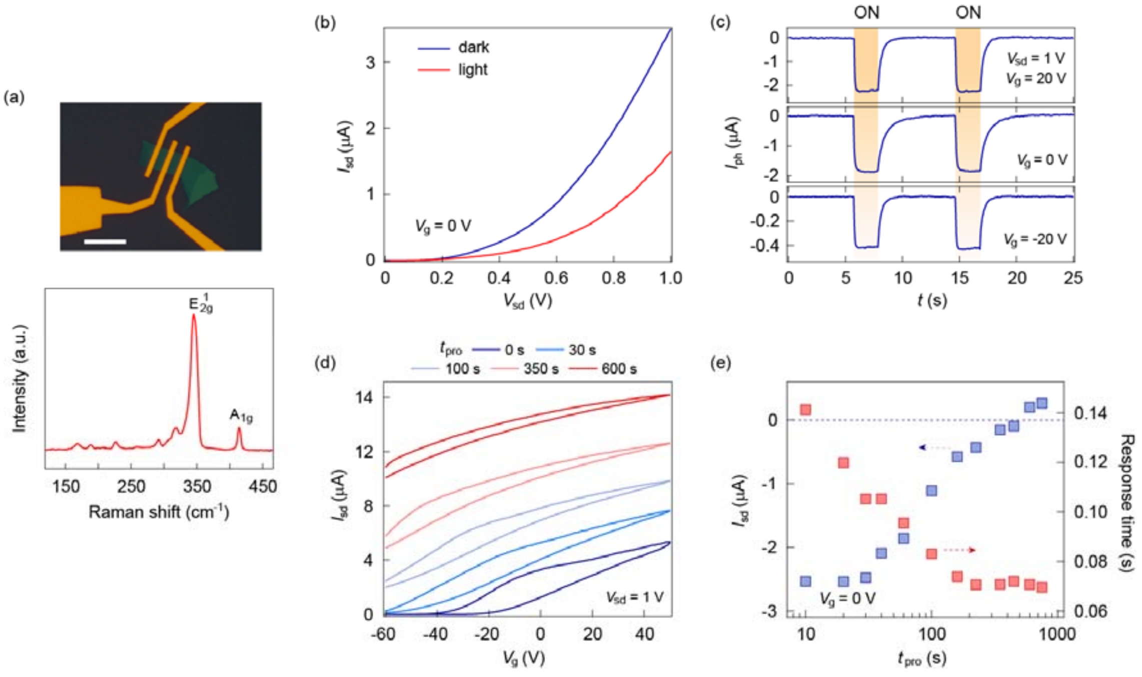

Figure S21 | Photodetection behavior of the WS_2/a-STO heterostructure and its evolution under light illumination. Scale bar is 5 μm.

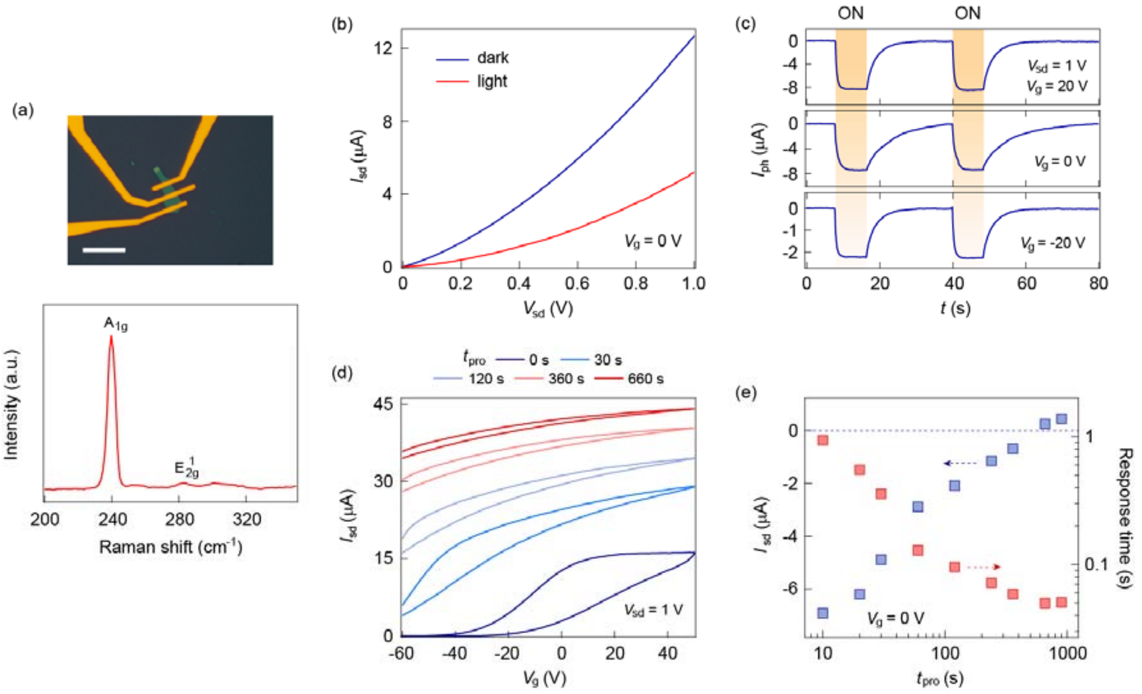

Figure S22 | Photodetection behavior of the $MoSe_2$/a-STO heterostructure and its evolution under light illumination. Scale bar is 5 μm.
